# Outgrowth Endothelial Cell Conditioned Medium Negates TNF-α-Evoked Cerebral Barrier Damage: A Reverse Translational Research to Explore Mechanisms

**DOI:** 10.1007/s12015-022-10439-4

**Published:** 2022-09-02

**Authors:** Rais Reskiawan A. Kadir, Mansour Alwjwaj, Kamini Rakkar, Othman Ahmad Othman, Nikola Sprigg, Philip M. Bath, Ulvi Bayraktutan

**Affiliations:** Academic Unit of Mental Health and Clinical Neuroscience, Clinical Sciences Building, School of Medicine, The University of Nottingham, Hucknall Road, Nottingham, NG5 1PB UK

**Keywords:** Endothelial progenitor cells, Blood–brain barrier, Secretome, Stem cells, Stroke, Reverse translational study

## Abstract

Improved understanding of the key mechanisms underlying cerebral ischemic injury is essential for the discovery of efficacious novel therapeutics for stroke. Through detailed analysis of plasma samples obtained from a large number of healthy volunteers (*n* = 90) and ischemic stroke patients (*n* = 81), the current study found significant elevations in the levels of TNF-α at baseline (within the first 48 h of stroke) and on days 7, 30, 90 after ischaemic stroke. It then assessed the impact of this inflammatory cytokine on an *in* *vitro* model of human blood–brain barrier (BBB) and revealed dramatic impairments in both barrier integrity and function, the main cause of early death after an ischemic stroke. Co-treatment of BBB models in similar experiments with outgrowth endothelial cell-derived conditioned media (OEC-CM) negated the deleterious effects of TNF-α on BBB. Effective suppression of anti-angiogenic factor endostatin, stress fiber formation, oxidative stress, and apoptosis along with concomitant improvements in extracellular matrix adhesive and tubulogenic properties of brain microvascular endothelial cells and OECs played an important role in OEC-CM-mediated benefits. Significant increases in pro-angiogenic endothelin-1 and monocyte chemoattractant protein-1 in OEC-CM compared to the secretomes of OEC and HBMEC, detected by proteome profiling assay, accentuate the beneficial effects of OEC-CM. In conclusion, this reverse translational study identifies TNF-α as an important mediator of post-ischemic cerebral barrier damage and proposes OEC-CM as a potential vasculoprotective therapeutic strategy by demonstrating its ability to regulate a wide range of mechanisms associated with BBB function. Clinical trial registration NCT02980354.

**Graphical Abstract:**

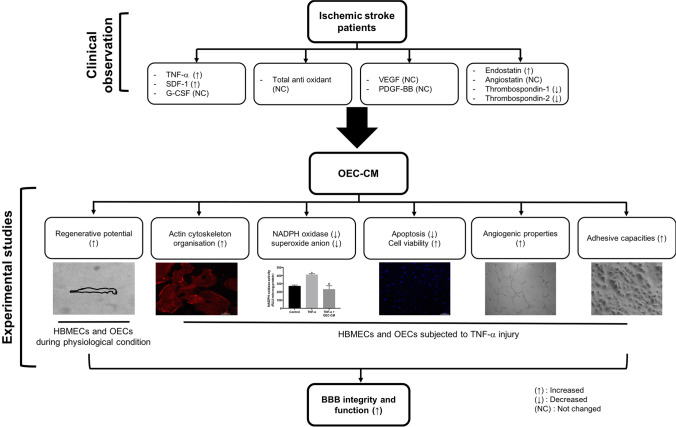

**Supplementary Information:**

The online version contains supplementary material available at 10.1007/s12015-022-10439-4.

## Introduction

Ischemic stroke (IS), stemming from an interference with blood supply to the brain, continues to be one of the leading causes of mortality and morbidity worldwide [1, 2]. Thrombolysis with intravenous recombinant tissue plasminogen activator (rtPA) and mechanical thrombectomy are the only approved treatment options for acute IS. However, due to short therapeutic windows associated with these treatments (up to 4.5 h and 24 h, respectively), each year < 1% of patients receive these therapies globally [3–5]. The complex pathophysiology of stroke involves many processes, including ATP depletion, excitotoxicity, inflammation, oxidative stress, necrosis, apoptosis and an increase in intracellular calcium levels [6, 7]. Hence, a better understanding of mechanisms involved in the pathogenesis and outcome of stroke is of paramount importance [8]. In this context, reverse translational studies, working from bedside to bench, may be of enormous help to uncover the main causative factors and help inform the design of future therapeutic strategies [9, 10]. Through detailed analysis of plasma samples of IS patients and healthy volunteers (HVs) recruited for The Dunhill Medical Trust EPC (DMT EPC) study, this study has identified inflammatory cytokine tumor necrosis factor-α (TNF-α) as an important factor for the progression and outcome of IS and showed significant elevations in its level during acute, subacute and chronic phases of the disease [11].

Again, considering that the majority of mortalities occur within the first week of an IS due to disruption of blood–brain barrier (BBB) and ensuing formation of cerebral edema, restoration of BBB function is now regarded as an important therapeutic priority [7, 12]. Endothelial progenitor cells (EPCs) and their functional subtype, called outgrowth endothelial cells (OECs), may make one such therapeutics. EPCs/OECs can differentiate into mature ECs and release a wide range of trophic factors to maintain BBB integrity in all (physio)pathological settings [13]. Indeed, recovery of BBB and neurological functions in stroke rats treated with OECs 24–48 h after middle cerebral artery occlusion (MCAo) confirmed the ability of these cells to engraft into brain capillaries and serve as therapeutics [14, 15]. However, replicative senescence of OECs during large scale *ex* *vivo* expansion and elevated risk of emboli formation and immune reaction associated with cell-based therapies have thus far hampered the widespread use of these particular cells in clinical practice [16].

As post-ischemic neurovascular reparative function of OECs is widely attributed to a wide range of factors, e.g. angiogenin, interleukin-8, platelet-derived growth factor-BB (PDGF-BB) and vascular endothelial cell growth factor (VEGF) that they secrete, OEC-derived conditioned medium (OEC-CM) has attracted attention as an alternative therapeutic [17, 18]. In addition to addressing most side effects associated with cell-based therapies, an OEC-CM-based therapeutic approach also offers benefits concerning the ease of production, handling, storage, and cost [17, 19].

In light of the above, using a well-established *in* *vitro* model of human BBB, this reverse translational study aims to investigate whether OEC-CM can protect BBB from the deleterious effects of TNF-α and attempts to delineate mechanisms involved in its putative beneficial effects.

## Materials and Methods

### Study Design

DMT EPC study followed the Standard Protocol Items: Recommendations for Interventional Trials (SPIRIT) guidance for protocol reporting. The study protocol was reviewed and approved by West Midlands—Coventry & Warwickshire Research Ethics Committee (16/WM/0304). Although details of the study have previously been described elsewhere, it is of note that individuals with ≥ 140/90 mmHg blood pressure, ≥ 7 mmol/L fasting glucose or 11.1 mmol/L 2 h post-prandial glucose level and with ≥ 5 mmol/L total cholesterol level were successively considered as hypertensive, diabetic and hyperlipidaemic [11].

In brief, the DMT EPC investigates the quantity and functionality of circulating EPCs as well as the biochemical profile of HVs and IS patients at four different timepoints following ischemic injury: days 0 (within 48 h of IS symptom onset), 7, 30, and 90, covering acute, subacute, and chronic phases of IS (Supplementary Fig. 1). To this end, 30 mL blood samples were taken from each participant at each timepoint and were split for the abovementioned objectives. The first 6 mL were used to quantify the number of circulating EPCs by flow cytometry, and the plasma was simultaneously extracted to assess the biochemical profiles of participants, while the remaining blood was used to produce OECs.

### Biochemical Assays

Specific ELISAs were used *as* *per* the manufacturers’ (all from R&D Systems unless otherwise stated) instructions to assess the plasma levels of TNF-α, granulocyte colony-stimulating factor (G-CSF), PDGF-BB, stromal derived factor-1 (SDF-1), total anti-oxidant capacity (Abcam), VEGF, endostatin, angiostatin (Abcam), thrombospondin-1, and thrombospondin-2.

### Cell Culture

Human brain microvascular endothelial cells (HBMECs), pericytes, and astrocytes were purchased from TCS CellWorks Ltd. (Buckingham, UK) and cultured at 37 °C in a humidified atmosphere (75% N_2_, 20% O_2_, 5% CO_2_) with their respective media (Sciencell Research Laboratories, San Diego, USA). Mononuclear cells isolated from a 24-ml of human peripheral blood sample were cultured on fibronectin-coated plates to encourage the growth of OECs as previously reported [13]. The Endothelial Basal Medium-2 (EBM-2, Lonza), containing 20% FBS and all the supplements provided with the medium, was used to culture OECs. Once obtained, OECs were characterized, as cells possessing both endothelial and progenitor cell properties, using a series of morphological, immunophenotypic, and tubulogenic assessments as before [13].

Due to consistent elevations observed in HBMEC oxidative stress and BBB permeability with 10 ng/mL TNF-α for 6 h, this particular timepoint and concentration were used throughout the current study [20].

### Acquisition of OEC-CM

OEC-CM was generated by culturing ~ 90% confluent OECs, isolated from the blood samples of three different healthy individuals, with EBM-2 containing 1% FBS but lacking all other supplements in hypoxic conditions (1.5% O_2_, 5% CO_2_, 93.5% N_2_) for 48 h [18]. This was followed by collection, centrifugation (10,000 rpm at 4 °C), filtration (via a 0.22 μm filter), and snap freezing of supernatant at -80 °C. EBM-2 containing 1% FBS and lacking all other supplements was used as a control medium.

### Proteome Profiling of Cells

A proteome profiler human angiogenesis array (R&D Systems) was used to assess the angiogenic factors released from the cells as per the manufacturer’s instructions. To generate OEC and HBMEC secretomes, cells were cultured under normal conditions (75% N_2_, 20% O_2_, 5% CO_2_) for 48 h in EBM-2 containing solely 1% FBS. OEC-CM, generated as above, were also used in the studies.

### Establishment of a Triple Cell Culture Model of BBB

For this, ~ 7.5 × 10^4^ human astrocytes (HAs) were seeded on the basal side of polyester Transwell inserts (0.4 µm pore size, 12 mm diameter polyester membrane, High Wycombe, UK). On the following day, the inserts were inverted the right way and placed into a 12-well plate containing fresh medium. Once astrocytes reached ~ 90% confluence, HBMEC (~ 5 × 10^4^ cells), alone or mixed with OECs (2:1 HBMEC:OEC ratio), were seeded onto the apical side of the inserts. When both cell layers were fully confluent, Transwell inserts were transferred to 12-well plates containing confluent human pericytes (HPs) to establish the triple cell culture model of human BBB [21].

### Assessment of BBB Integrity and Function

The BBB integrity and function were assessed as before by measurements of transendothelial electrical resistance (TEER, World Precision Instruments, Hertfordshire, UK) and paracellular flux of the low molecular weight permeability marker, sodium fluorescein (NaF, 376 Da), respectively [21].

### Measurement of NADPH Oxidase Capacity and Superoxide Anion Level

NADPH oxidase activity and the levels of superoxide anion were measured by lucigenin chemiluminescence and cytochrome-C reduction assays, respectively [22].

### Wound Scratch Assay

OECs and HBMECs (1.5 × 10^4^ cells/well) were seeded in 6-well plates with their specialized media until reaching 90% confluence. A scratch was then made by scraping the cell layer across each culture plate using the tip of a p1000 micropipette in one swift motion. The cells were washed with phosphate-buffered saline (PBS) to remove the debris. Pictures of wound closure were taken immediately after the scratch (0 h) and 24 h after this. Wound closure was quantified as the percentage of difference in scratch area between 0 and 24 h using ImageJ software (version 1.52 k, NIH, Maryland, USA).

### Proliferation Assay

Cellular proliferation rates were determined using the 4-[3-(4-lodophenyl)-2-(4-nitrophenyl)-2H-5-tetrazolio]-1.3-benzene disulfonate (WST-1) kit (Roche, Mannheim, Germany). For this, ~ 5 × 10^3^ cells, seeded and cultured in a 96-well plate for 48 h, were firstly exposed to the respective experimental conditions. 100 μL fresh medium replacement containing 10 μL of WST-1 was then added prior to a further 2 h incubation at 37 °C. The absorbance (450 nm) was subsequently read, using a FLUOstar Omega plate reader (BMG Labtech Ltd., UK).

### Tube Formation Assay

Firstly, growth factor-reduced Matrigel (50 μL/well, BD Biosciences) was added to 96-well plates and allowed to polymerize at 37 °C. 1 × 10^4^ cells were then seeded to each well and subjected to respective experimental conditions. The specific characteristics of the network of tubules, defined as the sum of number or length of segments, isolated elements and branches detected in the analyzed area, were then assessed using ImageJ software (version 1.52 k, NIH, Maryland, USA) [23].

### Cell Adhesion Assay

Equal number of cells, grown in 6-well plates and exposed to experimental conditions, were seeded on fibronectin (Sigma) or collagen (Sigma) pre-coated 96-well plates. Following 1 h incubation, the plates were gently washed with PBS and examined under light microscope. The adherent cells were counted in at least four random fields per well.

### Cell Viability and Caspase 3/7 Assay

Calcein AM (Calbiochem, Massachusetts, USA) and Apo-ONE homogeneous caspase-3/7 assay kit (Promega, Southampton, UK) were used to measure cell viability and caspase 3/7 activity, respectively as described before [24].

### Immunocytochemistry

The organization of actin cytoskeleton was studied by staining of F-actin filaments with rhodamine phallodin as before [25]. Cells grown on coverslips were fixed in 4% paraformaldehyde/PBS for 20 min and permeabilized with 0.1% Triton X-100/PBS for 15 min before staining with 1 × rhodamine phalloidin for 60 min (Abcam, Cambridge, UK). The coverslips were then mounted on glass slides prior to visualization with fluorescence microscopy (Zeiss Axio Observer, Carl Zeiss Ltd., Cambridge, UK). The number of stress fibres was manually counted.

### Detection of Apoptosis

Cells, subjected to different experimental conditions, were fixed in 4% paraformaldehyde/PBS for 20 min and stained for 5 min at room temperature with Hoechst 33,258 (10 μg/mL, Sigma). The coverslips were mounted on glass slides and analyzed by fluorescence microscopy (Zeiss Axio Observer, Carl Zeiss Ltd., Cambridge, UK).

### Statistical Analyses

The statistical analyses were performed either with GraphPad Prism 8.0 statistical software package (GraphPad Software Inc., La Jolla, Ca, USA) or SPSS package 15.0 for Windows (SPSS Inc., Chicago, Illinois, USA). Statistical tests were performed by *t*-test, Mann–Whitney, chi-square, or one-way analysis of variance followed by Tukey’s post hoc analysis, where appropriate. *P* < 0.05 was considered as significant.

## Results

### Patients with IS Display Significant Variations in Inflammatory and Angiogenic Modulator Generation

**Fig. 1:**
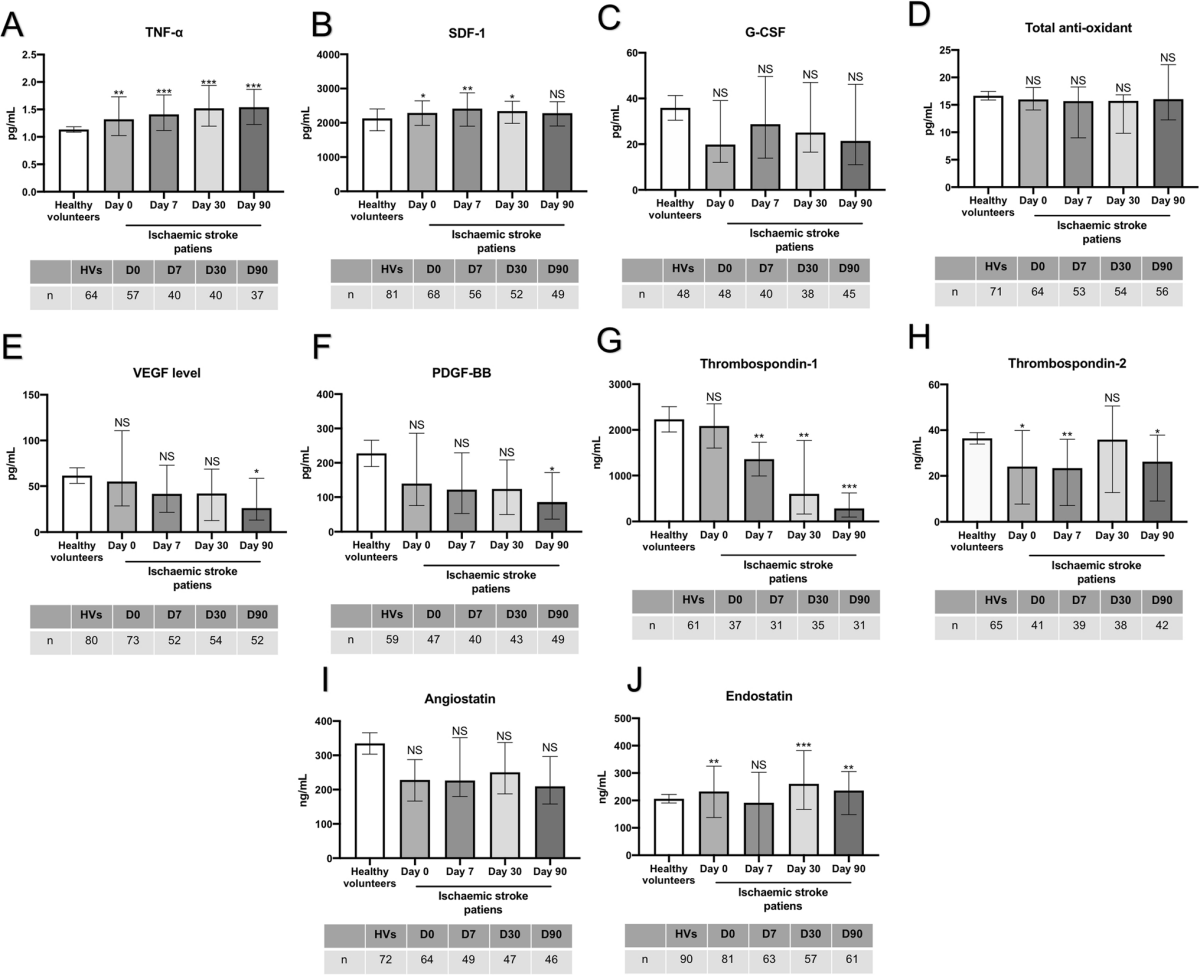
The plasma level of major elements that have relevance to endothelial function in patients with IS and HVs. **(A-D)** The levels of pro-inflammatory mediator TNF-α and chemoattractant SDF-1 increased in patients at different phases of ischemic stroke while no differences were observed in the levels of G-CSF and total antioxidant capacity. **(E, F)** Gradual decreases observed in pro-angiogenic factors, VEGF and PDGF-BB reached significance at day 90 of ischemic stroke. **(G-J)** Ischemic stroke differentially regulated the levels of angiogenic suppressors in that the levels of endostatin constantly increased after ischemic injury while the levels of thrombospondin-1, and thrombospondin-2 decreased. No change was observed in angiostatin levels. * < 0.05, **P < 0.01, ***P < 0.001 versus HVs (*t* test or Mann Whitney U test). NS: not significant compared to HVs. G-CSF, granulocyte colony-stimulating factor; HVs, healthy volunteers; IS, ischemic stroke; PDGF-BB, platelet-derived growth factor; SDF-1, stromal cell-derived factor 1; TNF-α, tumor necrosis factor-α; VEGF, vascular endothelial growth factor

A total of 90 HVs and 81 patients were recruited for the study. Table 1 summarizes the demographical and clinical data collected for all participants. Compared to HVs, IS patients had substantially higher TNF-α levels which remained elevated at all timepoints covered in the study (Fig. 1A). In light of the etiological differences between cortical and lacunar strokes, i.e. large artery disease and endothelial dysfunction, respectively, this study specifically assessed the levels of plasma TNF-α in both subgroups and found similar readings (Supplementary Fig. 2).

**Table 1:** Baseline characteristics of healthy volunteers and ischaemic stroke patients

	Healthy volunteers *n* = *90*	Patients *n* = *81*	*p* value	Cortical stroke *n* = 43	Lacunar stroke *n* = 38	*p* value
Age (years) median	61.5	76	< 0.0001	77.2	76.4	0.921
Male (*n*)	35	56	< 0.0001	27	29	0.188
Smoking (current smoker, *n*)	32	48	0.003	26	22	0.927
Alcohol ≥ 3 unit/week (*n*)	33	33	0.534	18	15	0.835
Hypertension (*n*)	21	49	< 0.0001	30	19	0.091
Diabetes (*n*)	9	17	0.038	8	9	0.365
AF (*n*)	3	23	< 0.0001	17	6	0.019
Hyperlipidemia (*n*)	15	24	0.061	14	10	0.542
TIA (*n*)	0	15	< 0.0001	4	11	0.024
IS (*n*)	0	12	< 0.0001	8	4	0.310
ICH (*n*)	0	0	-	0	0	-
CAD (*n*)	2	4	0.337	2	2	0.90
PAD (*n*)	0	2	0.135	1	1	0.930
Statins (*n*)	21	38	0.001	22	16	0.482
Ace inhibitor (*n*)	16	34	< 0.0001	22	12	0.093
CCB (*n*)	8	17	0.023	14	3	0.008
Beta blocker (*n*)	6	15	0.017	9	6	0.592
Nitrates (*n*)	2	3	0.556	2	1	0.649
Anti-platelet (*n*)	7	33	< 0.0001	18	15	0.905
Insulin (*n*)	1	2	0.492	1	1	0.915
Glucose lowering	6	13	0.048	4	19	0.07
Exercise (moderate-vigorous) (*n*)	55	26	< 0.0001	13	13	0.679
NIHSS on admission (mean)	-	4.85	-	5.63	3.95	0.002
mRS ≤ 2 (*n*)	-	56 (82.4%)	-	30 (81%)	26 (82.8%)	0.271
BI ≥ 90 (*n*)	-	59 (91.2%)	-	31 (93.9%)	28 (90.3%)	0.548

*AF* atrial fibrillation, *BI* Barthel index, *CAD* coronary artery disease, *CCB* calcium channel blocker, *ICH* intracerebral hemorrhage, IS ischemic stroke, *mRS* modified ranking score, *NIHSS* national institutes of health stroke scale, *PAD* peripheral arterial disease*,* *TIA* transient ischaemic attack

Similarly, the level of chemokine SDF-1, known to mediate trafficking and homing of EPCs to the site of injury, was also higher in patients which peaked on day 7, gradually decreased from then on and normalized on day 90 (Fig. 1B). In contrast, no changes in the plasma level of cytokine G-CSF, known to mobilize EPCs, were observed between patients and HVs at any timepoint (Fig. 1C).

Oxidative stress, emerging from an imbalance between endogenous pro-oxidants and anti-oxidants, is thought to profoundly affect the pathogenesis and outcome of IS [26]. Analyses of the plasma total anti-oxidant capacity, determined by the sum of food-derived and endogenous anti-oxidants including the enzymes, small molecules and proteins (e.g. catalase, ascorbate, and albumin), have shown insignificant decreases in IS patients compared to HVs (Fig. 1D).

Effective neovascularization of infarct area, mediated by release of pro-angiogenic factors in the injured tissue, is crucial for recovery following an IS [27]. Gradual decreases in plasma levels of major pro-angiogenic factors, VEGF and PDGF-BB, reaching significance on day 90 after stroke compared to HVs, may in part explain the differences in stroke patients’ outcome (Fig. 1E, F). Interestingly, plasma levels of anti-angiogenic factors thrombospondin-1 and thrombospondin-2 also substantially decreased following IS (Fig. 1G, H). Insignificant decreases in plasma level of anti-angiogenic factor angiostatin were also observed in IS patients at all timepoints compared to HVs (Fig. 1I). In contrast, the level of endostatin significantly increased during the acute and chronic phases of stroke (Fig. 1J).

Since most IS patients have at least one vascular risk factor [28], this study examined the specific correlation between the number of vascular risk factors that patients had and their plasma TNF-α levels. Observation of similar TNF-α levels in patients with and without risk factors, other than those with DM on day 7 and with TIA at baseline, refuted this hypothesis and proposed ischemic injury as the main inducer of TNF-α in stroke patients (Supplementary Fig. 3A-E). However, the presence of multiple risk factors slightly elevated TNF-α levels which reached significance when DM was one of the risk factors (Supplementary Fig. 4A-D).

Detection of higher TNF-α levels in HVs with DM, hypertension and hyperlipidemia on the other hand, proved these risk factors as the generators of TNF-α in the absence of an overt ischemic injury (Supplementary Fig. 3F-I). However, co-existence of hypertension and diabetes mellitus increased TNF-α release compared to either group alone (Supplementary Fig. 4E).

### OEC-CM Protects BBB Integrity and Function Against TNF-α Injury

**Fig. 2:**
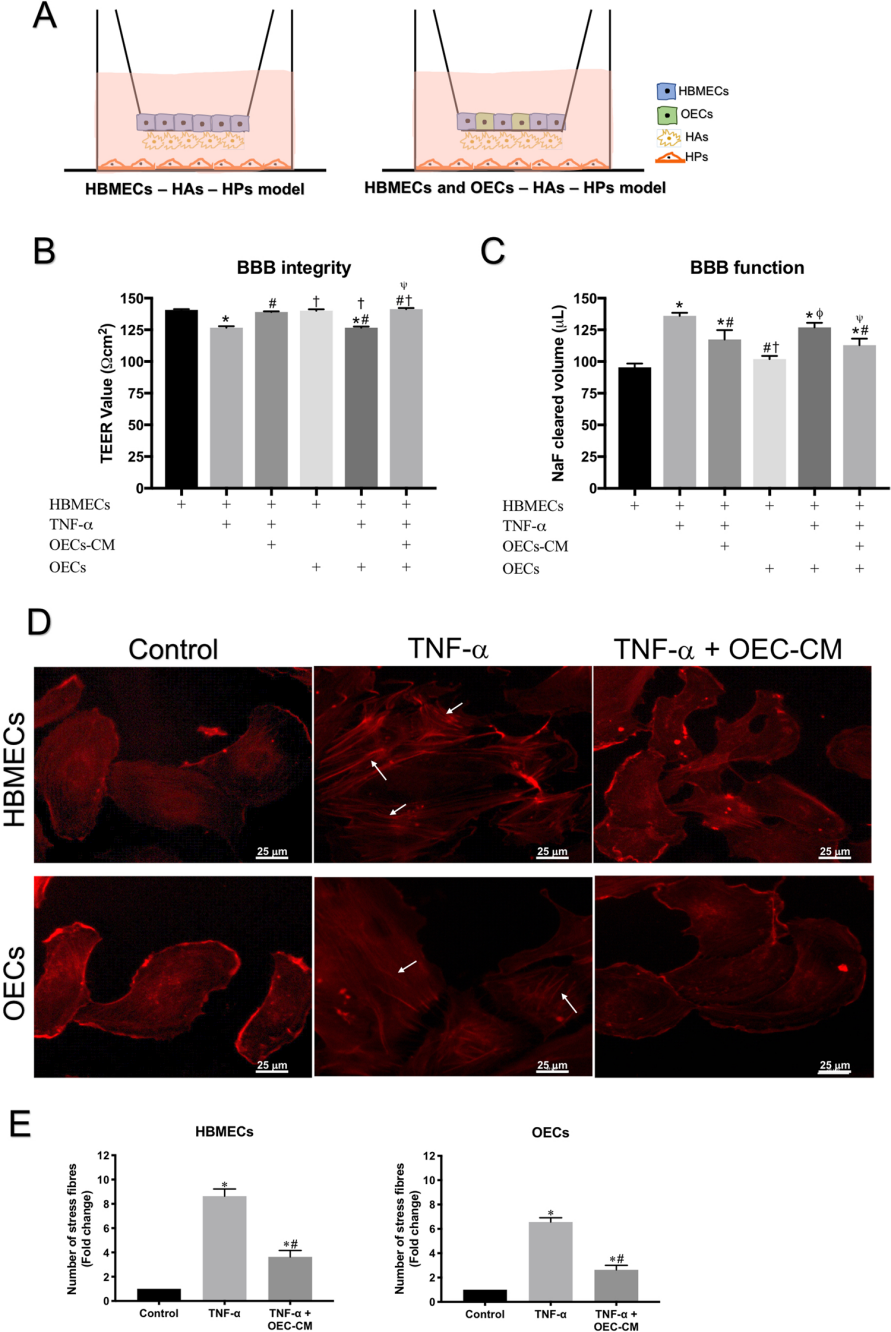
Schematic diagram of an *in* *vitro* model of human BBB and the effect of OEC-CM on BBB integrity and function and actin cytoskeleton organization in HBMECs and OECs. **(A)** *In* *vitro* models of human BBB consisting of astrocytes, pericytes, and HBMECs alone or mixed with OECs. **(B, C)** TNF-α significantly disrupted BBB integrity and function, as shown by decreases in TEER and concomitant increases in paracellular flux of sodium fluorescein, which were prevented by OEC-CM treatment. **(D)** Co-treatment with OEC-CM prevented the effects of TNF-α on cytoskeletal reorganization in HBMECs and OECs and decreased stress fiber formation (white arrows). **(E)** Quantification of stress fiber formation in both cells. Scale bar: 25 μm. *P < 0.05 versus BBB formed by HBMECs or control, ^#^*P* < 0.05 versus BBB formed by HBMECs exposed to TNF-α, ^†^*P* < 0.05 versus BBB formed by HBMECs exposed to TNF-α and OEC-CM, ^φ^*P* < 0.05 versus BBB formed by HBMECs and OECs, ^ψ^*P* < 0.05 versus BBB formed by HBMECs and OECs exposed to TNF-α (one-way ANOVA followed by Tukey's post-hoc analysis). BBB, blood–brain barrier; HBMECs, human brain microvascular endothelial cells; OEC-CM, outgrowth endothelial cell-derived conditioned medium; OECs, outgrowth endothelial cells; TNF-α, tumor necrosis factor-α

Increased TNF-α levels in patients and HVs with vascular risk factors strongly support the involvement of this cytokine in endothelial dysfunction, a prominent cause of BBB permeability [20, 29]. As cerebral edema constitutes the main cause of death after IS [7, 12], this study assessed the therapeutic potential of an emerging strategy, employing hypoxia-primed OEC-CM, in mitigating the previously reported barrier-disruptive effects of TNF-α. For this, two triple cell culture models of human BBB, consisting of astrocytes, pericytes, and HBMECs alone or mixed with OECs (Fig. 2A) were subjected to TNF-α (10 ng/ml) for 6 h in the absence or presence of OEC-CM. Reversal of TNF-α-induced decreases in TEER readings and increases in paracellular flux of NaF evinced the protective effect of OEC-CM on barrier integrity and function, respectively (Fig. 2B, C). Suppression of TNF-α-induced contractile actin bundles, otherwise known as stress fibers, leading to the formation of intercellular gaps between adjacent ECs [20, 30], also contributed to the barrier-protective role of OEC-CM (Fig. 2D, E). Scrutiny of mechanisms underlying the barrier-restorative effects of OEC-CM attributed these benefits to markedly reduced oxidative stress and apoptosis, ascertained by decreased NADPH oxidase activity, superoxide anion generation, DNA fragmentation, caspase 3/7 activity and increased cell viability in both HBMEC and OECs (Supplementary Figs. 5 and 6, respectively).

### OEC-CM Promotes Cellular Mechanisms Required for Angiogenesis

**Fig. 3:**
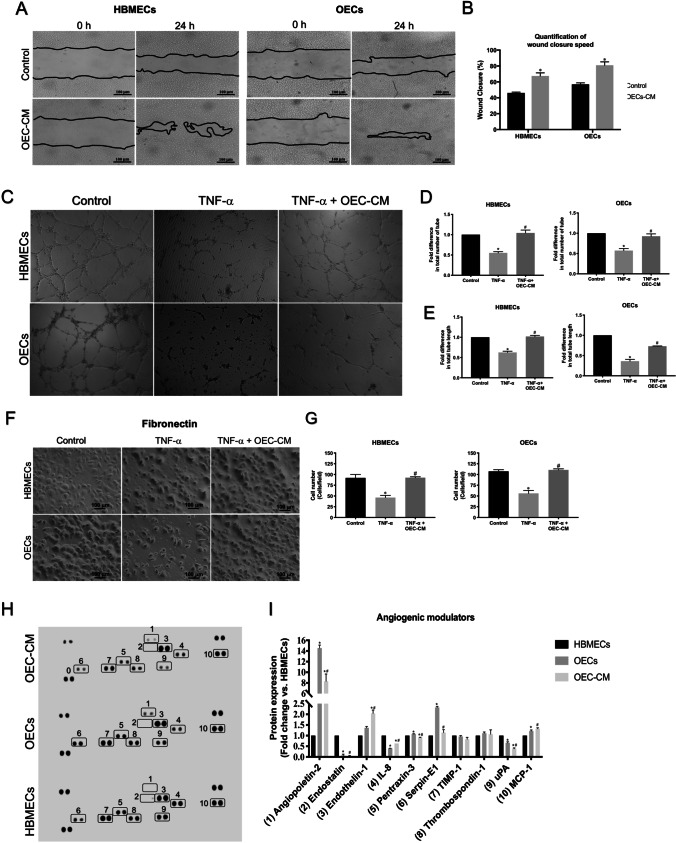
The effect of OEC-CM on HBMEC and OEC functional characteristics and analysis of angiogenesis-related proteins in HBMEC and OEC secretomes and OEC-CM. **(A, B)** OEC-CM accelerated wound closure in both HBMEC and OECs. **(C-E)** OEC-CM negated the impact of TNF-α on HBMEC and OEC tubule network. **(F, G)** Treatments with OEC-CM neutralized the inhibitory effect of TNF-α on HBMEC and OEC adhesion to fibronectin, an extracellular matrix protein. **(H, I)** Proteome profiling of OEC-CM along with HBMEC and OEC secretomes revealed significant variations in various pro- and anti-angiogenic factors e.g. endothelin-1, MCP-1 and endostatin in OEC-CM. Scale bars = 100 μm. *P < 0.05 versus control, ^#^*P* < 0.05 versus TNF-α (one-way ANOVA followed by Tukey's post-hoc analysis). HBMECs, human brain microvascular endothelial cells; IL-8, interleukin-8; MCP-1, monocyte chemoattractant protein-1; OEC-CM, outgrowth endothelial cell-derived conditioned medium; OECs, outgrowth endothelial cells; TIMP-1, tissue inhibitors of metalloproteinase-1; TNF-α, tumor necrosis factor-α; uPA, urokinase plasminogen activator

Angiogenesis, formation of new blood vessels from pre-existing ones, is an important process in post-ischemic tissue injury and BBB remodeling and involves the migration, proliferation, and differentiation of ECs [27, 31]. It is controlled by a number of specific factors, including inflammatory cytokines and adhesion molecules [32]. Considering these and the pivotal roles of BMECs and OECs in BBB formation, this study assessed the proliferative and migratory potential of these particular cell lines in the absence and presence of OEC-CM and documented significant increases in both parameters in both cell types within 24 h of incubation using a wound scratch (Fig. 3A, B) and WST-1 assay (Supplementary Fig. 7).

The formation of tubules by ECs constitutes another critical step in the angiogenic process which occurs during growth of the incipient vascular sprout and requires the synchronized functioning of cytoskeleton and cell–matrix adhesion [33–35]. By nullifying the disruptive effects of TNF-α on the number and length of tubes formed on Matrigel, a reliable marker for angiogenesis in vivo [36], OEC-CM may promote help revascularize the ischemic tissue (Fig. 3C-E). Similarly, OEC-CM abrogates the disruptive effects of TNF-α on cell-extracellular matrix adhesion, a prerequisite for migration, proliferation, and tubulogenic activity, as evidenced by dramatic increases in the number of HBMEC and OECs adhering to fibronectin (Fig. 3F, G) and collagen (Supplementary Fig. 8A, B), important components of the ECM, in equal measures.

Proteome profiling of OEC-CM versus HBMEC and OEC secretome in an attempt to identify the specific elements involved in aforementioned OEC-CM-mediated benefits revealed significant increases in pro-angiogenic factors, endothelin-1 (ET-1) and monocyte chemoattractant protein-1 (MCP-1) and a decrease in anti-angiogenic factor endostatin in OEC-CM (Fig. 3H, I). ET-1 stimulates the mobilization and proliferation of ECs through activation of both ETA and ETB receptors [37, 38] where specific activation of ETA receptor increases VEGF expression which in turn directly or through induction of MCP-1 promote EC migration and proliferation and participate in angiogenesis [39–41].

Compared to OECs, a specific increase in interleukin-8 (IL-8) and decreases in urokinase plasminogen activator (uPA), serpin-E1, angiopoietin-2 (Ang2), and pentraxin-3 levels were also found in OEC-CM. Like endothelins, IL-8 also directly enhances EC proliferation and survival [42]. Serpin-E1 or serine protease inhibitor-1 acts as the main inhibitor of uPA, an enzyme that catalyzes the conversion of plasminogen to plasmin. Attenuation of uPA in hypoxia-induced OEC-CM may maintain normal cellular phenotype and help prevent vascular leakage through regulation of MMP-2 and NADPH oxidase [43–45]. Similarly, Ang2 and acute phase protein pentraxin-3 also play critical roles in angiogenesis and vascular dysfunction in conditions associated with TNF-α release [46]. Observation of similar levels of thrombospondin-1 and tissue inhibitor of metalloproteinase-1 (TIMP-1) in OEC-CM and HBMEC and OEC supernatants rule out the possible involvement of these proteins in OEC-CM-mediated cell proliferation and migration (Fig. 3H, I).

Given the conspicuous differences in HV and IS patient plasma endostatin levels and the role of this anti-angiogenic factor in EPC functionality and post-stroke angiogenesis [47, 48], this study specifically assessed the impact of OEC-CM on endostatin levels in HBMEC and OEC subjected to TNF-α and significantly inhibited TNF-α-mediated elevations observed in both cell types (Supplementary Fig. 9).

## Discussion

Despite introduction of various guidelines, such as ARRIVE and STEPS [49, 50] to facilitate extrapolation of pre-clinical data to clinical settings, most stroke trials have failed to replicate the favorable results observed in relevant pre-clinical studies [8]. Scrutiny of the past failures suggest that critical analysis of clinical and biochemical changes brought about by ischemic injury may help identify new therapeutic targets and inform the design of pre-clinical research [10]. Through assessment of a large set of clinical, demographic, and biochemical data obtained from the participants of the DMT EPC study, the current study has found a consistent increase in TNF-α levels across all phases of IS compared to HVs. Similar increases have also been found in SDF-1 levels during the acute, subacute, and early chronic phase of IS. As SDF-1 mediates mobilization and homing of EPCs to the site of injury, its increased release in the immediate aftermath of stroke may counter the disruptive effects of stroke on cerebrovasculature, reduce infarct volume, and improve functional outcome [51]. Because cell damage and inflammation are mostly returned to normal levels in the later stages of stroke, SDF-1 may not be required as much, explaining the gradual decreases observed in its levels during the chronic phases of stroke.

Unlike TNF-α and SDF-1, no significant variation was observed in the levels of VEGF and PDGF-BB between IS patients and HVs until day 90 post-stroke. Despite presence of reports showing dramatic increases in serum VEGF levels in IS patients [52, 53], the findings of recent studies and a meta-analysis analyzing data from 14 case–control studies concur with the findings of the present study and indicate a lack of correlation between serum VEGF or PDGF-BB levels and time of testing performed after IS [54–56]. Even so, future multicenter studies with bigger and heterogenous participant population may better address these points.

Due their ability to re-endothelialize damaged endothelium and promote angiogenesis, EPCs and their functional subtype OECs have recently been at the forefront of clinical investigation [12, 57]. Subsequent studies have attributed much of the vasculoreparative effects of EPCs/OECs to the trophic factors that they release [58]. Using an interactive *in* *vitro* model of human BBB, the present study has shown that treatment with hypoxia-primed OEC-CM, a promising cell-based but cell-free strategy, mitigates the disruptive effects of TNF-α on vascular integrity and function, as proven by increases in TEER readings and decreases in paracellular flux of NaF. However, the capacity of EPCs to replace the dead or damaged cerebral ECs to maintain vascular homeostasis cannot be overlooked [12, 13]. Indeed, establishment of equally functional BBB with HBMECs alone or in combination with OECs confirm the ability of OECs to interact with mature ECs.

Enhanced NADPH oxidase activity and O_2_^.−^ production are involved in TNF-α-induced BBB damage [20, 29]. Observation of similar TAC in IS patients across all study timepoints indicate that pro-oxidant, rather than anti-oxidant, pathways plays a major role following cerebral ischemia. Diminished BBB damage and infarct volumes in NADPH oxidase-knockout versus wild-type mice and those treated with NADPH oxidase inhibitors (apocynin or DPI) before induction of MCAo, confirm the barrier-disruptive impact of this oxidase in IS settings [59, 60]. Inhibition of NADPH oxidase activity and O_2_^.−^ production in HBMECs and OECs subjected to TNF-α with OEC-CM prove the anti-oxidant effect of this therapy. Suppression of ROS, apoptosis, and inflammatory mediator generation in ECs subjected to H_2_O_2_ by OEC-CM reinforces the modulatory role of this therapeutic regimen on pro-oxidative mechanisms [61].

Apoptosis of cerebrovascular cells induced by inflammatory mediators may also contribute to cerebral barrier damage [30]. Analyses of several markers for cell viability/death have shown significant elevations in DNA fragmentation, pro-apoptotic caspase-3/7 enzyme activities and cell death (ascertained by reduced calcein AM staining) in TNF-α-treated HBMECs and OECs. Increased TUNEL (terminal deoxynucleotidyl transferase dUTP nick end labeling) staining, capable of detecting DNA breaks in the latter phases of apoptosis, had also previously been shown in HBMECs exposed to TNF-α [30]. Co-treatment of HBMEC and OECs with OEC-CM in the current study attenuated the apoptotic effects of TNF-α, markedly increased cell viability and invariably helped maintain appropriate BBB function. Treatment of oligodendrocyte precursor cells subjected to oxygen–glucose deprivation with EPC-CM has previously been shown to inhibit apoptosis [62]. Activation of canonical NF-κB pathway that regulates expression of various genes related to cell survival, proliferation, differentiation and inflammatory responses may also be instrumental in TNF-α-evoked barrier disruption. However, treatments with EPC secretome has been shown to suppress NF-κB transcriptional activity, leading to enhanced neovascularization *in* *vivo* following stroke and myocardial ischemia and restoring BBB integrity *in* *vitro* [63–65].

In addition to reparative processes, regenerative processes, notably angiogenesis involving EC proliferation, tubulogenesis, branching and anastomosis also contribute to neurological recovery after IS [27]. Progressive declines in plasma level of pro-angiogenic VEGF and PDGF-BB and a concomitant increase in anti-angiogenic endostatin observed in this study appear to be the main drivers of impaired angiogenesis after IS. Furthermore, the increased tubule network dissolution and impaired proliferation concurred with endostatin overproduction in HBMECs and OECs treated with TNF-α affirmed the seminal role of this cytokine in vitiated angiogenesis [66]. Normalization of these elements by OEC-CM displayed its modulatory influence on anti-angiogenic factor synthesis/release and affirmed its reparative function against TNF-α. Increased adhesion of resident ECs and OECs to fibronectin and collagen may also be instrumental in angiogenesis-stimulatory effect of OEC-CM, as strong adhesion to extracellular matrix components is an important prerequisite for the homing of circulating EPCs [67]. Neutralization of TNF-α-mediated activation of matrix metalloproteinases by OEC-CM is likely to contribute to its pro-angiogenic effects [68]. Detailed analysis of the elements released by OECs upon hypoxic-priming implicate ET-1, MCP-1, and IL-8 in the angiogenesis-promoting function of OEC-CM. Diminished expression of uPA, serpin-1, Ang2, endostatin, and pentraxin-3 may also be responsible for hypoxia-augmented pro-angiogenic effects of OEC-CM.

There are some limitations to this study. Since 88.2% of patients had mRS score of ≤ 3 on day 90 after stroke, the correlation between TNF-α levels and disease outcome could not be ascertained. As lacunar and cortical strokes made up almost half of the overall sample size, the neurological improvements was unlikely to derive from a particular stroke subtype. Considering that treatment with an anti-TNF-< antibody negate the ischemic injury-evoked BBB damage [29], analyses of barrier-restorative capacities of aTNF-α targeting agent, OEC-CM and combination of both would have been useful. It is likely that inflammatory mediators other than TNF-α, e.g. IL-6 and IL-33, may also significantly contribute to ischemic injury-evoked cerebral barrier damage [69, 70]. The specific roles of these inflammatory mediators and crosstalk between them during/after IS deserve attention in future studies.

## Conclusion

In depth analysis of plasma samples collected for The Dunhill Medical Trust EPC study has shown a constant increase in TNF-α levels in patients with IS compared to HVs. *In* *vitro* studies performed with a clinically-relevant concentration of TNF-< has proven that TNF-< compromises BBB integrity through a series of mechanisms, including EC apoptosis, oxidative stress, cytoskeletal reorganization and cell–matrix adhesion. Scrutiny of OEC-CM as an emerging cell-free therapeutic strategy has proven it to be very effective in negating the deleterious effects of TNF-< on BBB. Specific molecule(s) that contribute the most to the BBB-restorative effect of OEC-CM deserve further investigation in future studies. Similarly, it will be important to determine the therapeutic impact of OEC-CM in future *in* *vivo* studies.

## Supplementary Information

Below is the link to the electronic supplementary material.

Supplementary Figure captions (DOCX 16 KB)

Supplementary Fig. 1 (JPG 167 KB)

Supplementary Fig. 2 (JPG 46 KB)

Supplementary Fig. 3 (JPG 785 KB)

Supplementary Fig. 4 (JPG 96 KB)

Supplementary Fig. 5 (JPG 230 KB)

Supplementary Fig. 6 (JPG 3687 KB)

Supplementary Fig. 7 (JPG 23 KB)

Supplementary Fig. 8 (JPG 502 KB)

Supplementary Fig. 9 (JPG 21 KB)

## Data Availability

The datasets generated during and/or analysed during the current study are available from the corresponding author on reasonable request.
