# Supplementary material for: Outgrowth Endothelial Cell Conditioned Medium Negates TNF-α-Evoked Cerebral Barrier Damage: A Reverse Translational Research to Explore Mechanisms
Source: Stem Cell Rev Rep. 2022 Sep 2;19(2):503–15. doi: 10.1007/s12015-022-10439-4 (PMC9902316; doi:10.1007/s12015-022-10439-4)
Supplement: Supplementary file 1 — Supplementary Figure captions (DOCX 16 KB) [file 12015_2022_10439_MOESM1_ESM.docx]

**Supplementary fig. 1.** Study recruitment and patient pathway.

**Supplementary fig. 2. Levels of TNF-α in ischemic stroke patients.** The plasma level of TNF-α was similar in patients with cortical and lacunar stroke. NS: not significant compared to cortical stroke (*t* test or Mann Whitney U test).

**Supplementary fig. 3. Levels of TNF-α in ischemic stroke patients and healthy volunteers with and without comorbidities. (A-E)** The plasma levels of TNF-α in stroke patients with and without hypertension, DM, hyperlipidemia, AF and TIA remained similar in all experimental groups at all time points studied other than those with DM on day 7 and with TIA on admission where substantial decreases were observed. **(F-I)** In contrast, level of TNF-α was significantly higher in HVs with hypertension, DM, and dyslipidemia than those without. **P*<0.05, ***P*<0.01, ****P*<0.001 versus history (-) (*t* test or Mann Whitney U test). NS: not significant compared to without history. AF, atrial fibrillation; DM, diabetes mellitus; HVs healthy volunteers; IS, ischemic stroke; TIA, transient ischemic attack; TNF-α, tumor necrosis factor-α.

**Supplementary fig. 4. Levels of TNF-α in stroke patients and healthy voliunteers with several risk factors. (A-D)** The plasma level of TNF-α increased selectively in ischaemic stroke patients with DM and one or two additional risk factors i.e. HT and HL solely on days 0, 7 and 30. **(E)** In healthy volunteers, the level of TNF-α was only significantly higher in HT+DM. **P*<0.05 versus HT, ^#^*P*<0.05 versus HL, ^†^*P*<0.05 versus HT+HL, ^φ^*P*<0.05 versus DM+HL (*t* test or Mann Whitney U test). DM, diabetes mellitus; HT, hypertension; HL, hyperlipidemia; TNF-α, tumor necrosis factor-α.

**Supplementary fig. 5. Treatment with OEC-CM suppresses oxidative stress. (A-D)** TNF-α enhanced NADPH oxidase activity and superoxide anion level in both HBMEC and OECs which were markedly reduced by treatments with an OEC-CM. **P*<0.05 versus control, ^#^*P*<0.05 versus TNF-α (one-way ANOVA followed by Tukey's post-hoc analysis). HBMECs, human brain microvascular endothelial cells; OEC-CM, outgrowth endothelial cell-derived conditioned medium; OECs, outgrowth endothelial cells; TNF-α, tumor necrosis factor-α.

**Supplementary fig. 6. The impact of OEC-CM on HBMEC and OEC apoptosis and viability. (A-C)** Representative images and associated graphs showing the degree of nuclear degradation (as indicated by white arrows) in HBMEC and OECs treated with TNF-α in the absence or presence of OEC-CM. **(D-G)** OEC-CM also negated the TNF-α-evoked elevations in pro-apoptotic caspase-3/7 enzyme activities and improved their viability. Scale bars = 100 μm. **P*<0.05 versus control, ^#^*P*<0.05 versus TNF-α (one-way ANOVA followed by Tukey's post-hoc analysis). HBMECs, human brain microvascular endothelial cells; OEC-CM, outgrowth endothelial cell-derived conditioned medium; OECs, outgrowth endothelial cells; TNF-α, tumor necrosis factor-α.

**Supplementary fig. 7. The impact of OEC-CM on cellular proliferation.** TNF-α suppressed the proliferation rates of both HBMEC and OEC which were negated by OEC-CM. **P*<0.05 versus control, ^#^*P*<0.05 versus TNF-α (one-way ANOVA followed by Tukey's post-hoc analysis). HBMECs, human brain microvascular endothelial cells; OEC-CM, outgrowth endothelial cell-derived conditioned medium; OECs, outgrowth endothelial cells; TNF-α, tumor necrosis factor-α.

**Supplementary fig. 8. The effect of OEC-CM on cellular adhesion capacities.** TNF-α suppressed the adhesion of HBMEC and OEC to extracellular matrix protein, collagen which were negated by OEC-CM **(A-B)**. Scale bars = 100 μm. **P*<0.05 versus control, ^#^*P*<0.05 versus TNF-α (one-way ANOVA followed by Tukey's post-hoc analysis). HBMECs, human brain microvascular endothelial cells; OEC-CM, outgrowth endothelial cell-derived conditioned medium; OECs, outgrowth endothelial cells; TNF-α, tumor necrosis factor-α.

**Supplementary fig. 9. The impact of OEC-CM on endostatin levels.** TNF-α elevated the levels of endostatin, an angiogenesis inhibitor in both HBMEC and OECs. Treatments with OEC-CM effectively attenuated these increases in both cells. **P*<0.05 versus control, ^#^*P*<0.05 versus TNF-α (one-way ANOVA followed by Tukey's post-hoc analysis). HBMECs, human brain microvascular endothelial cells; OEC-CM, outgrowth endothelial cell-derived conditioned medium; OECs, outgrowth endothelial cells; TNF-α, tumor necrosis factor-α.
