# Supplementary figures and images for: Outgrowth Endothelial Cell Conditioned Medium Negates TNF-α-Evoked Cerebral Barrier Damage: A Reverse Translational Research to Explore Mechanisms
Source: Stem Cell Rev Rep. 2022 Sep 2;19(2):503–15. doi: 10.1007/s12015-022-10439-4 (PMC9902316; doi:10.1007/s12015-022-10439-4)

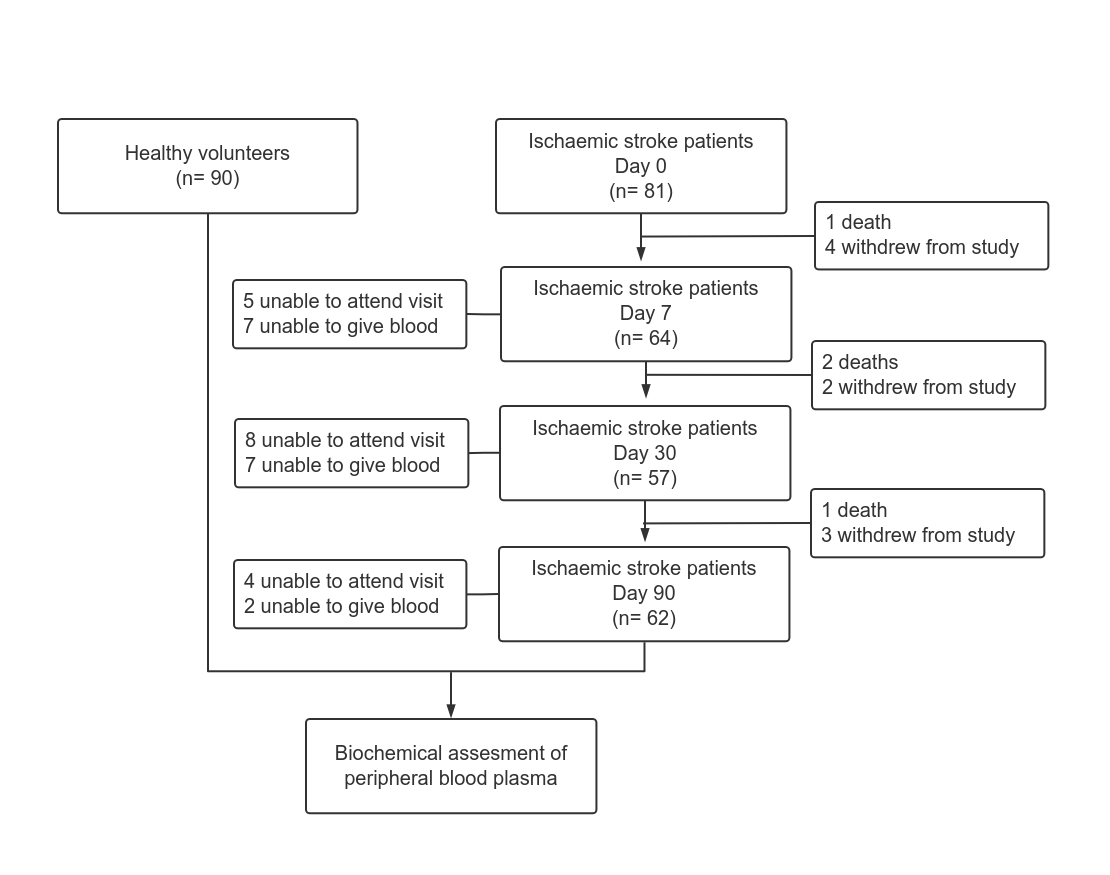

Supplement: Supplementary file 2 — Supplementary Fig. 1 (JPG 167 KB) [file 12015_2022_10439_MOESM2_ESM.jpg]

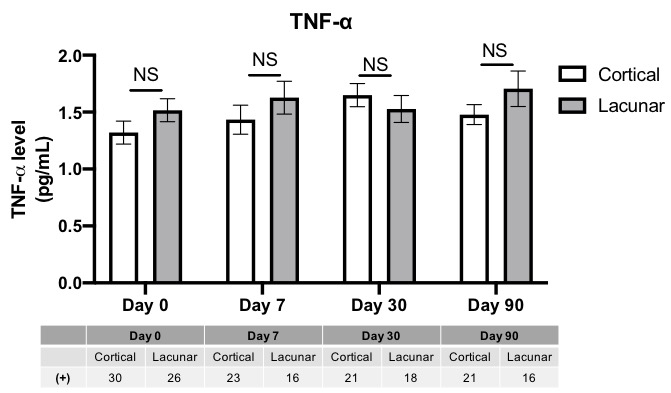

Supplement: Supplementary file 3 — Supplementary Fig. 2 (JPG 46 KB) [file 12015_2022_10439_MOESM3_ESM.jpg]

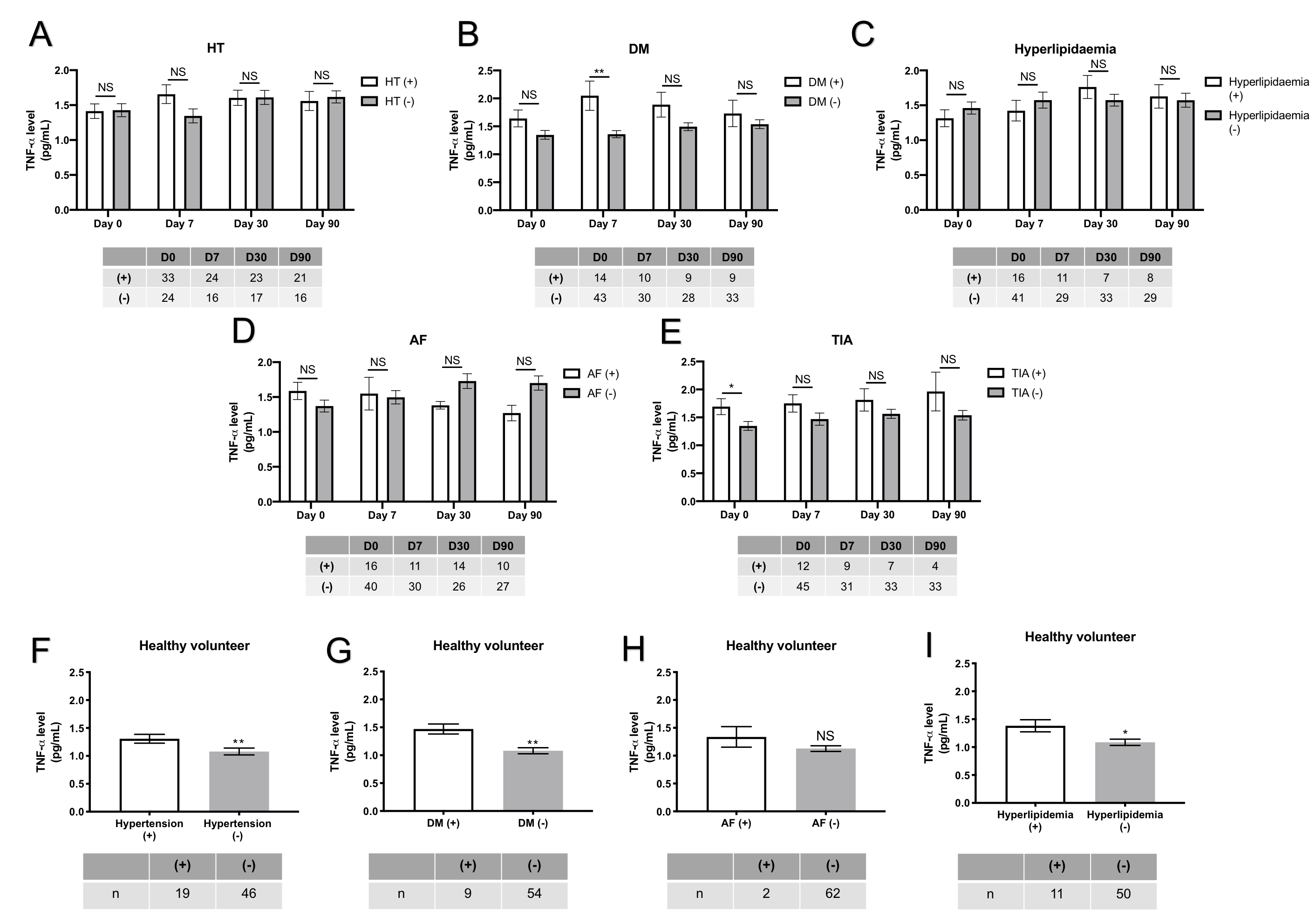

Supplement: Supplementary file 4 — Supplementary Fig. 3 (JPG 785 KB) [file 12015_2022_10439_MOESM4_ESM.jpg]

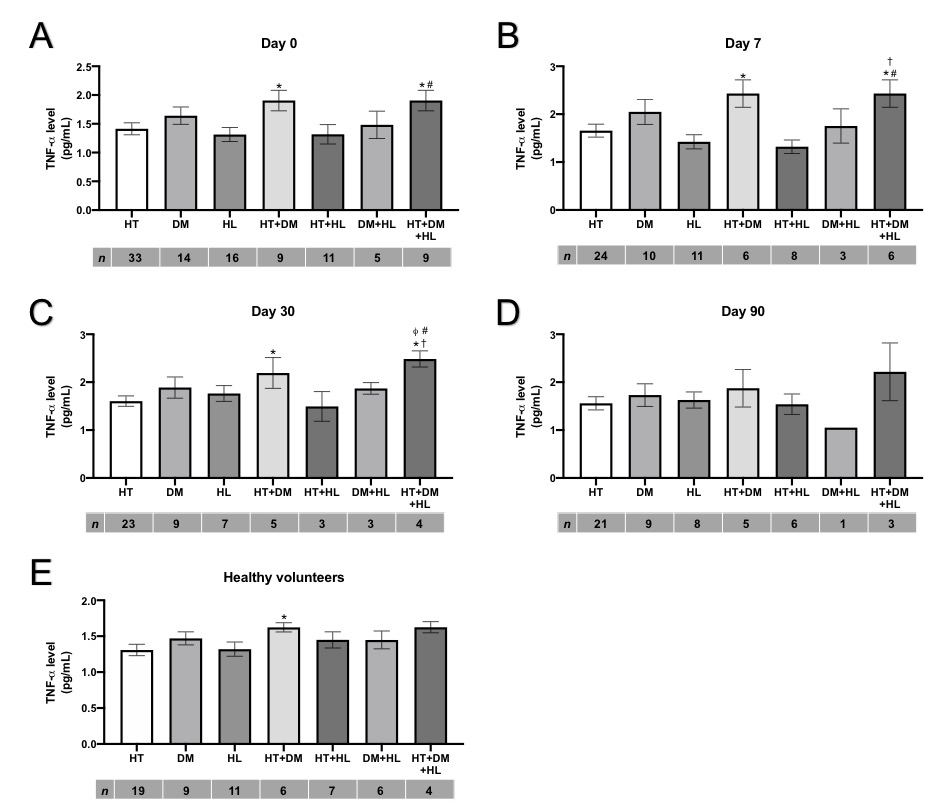

Supplement: Supplementary file 5 — Supplementary Fig. 4 (JPG 96 KB) [file 12015_2022_10439_MOESM5_ESM.jpg]

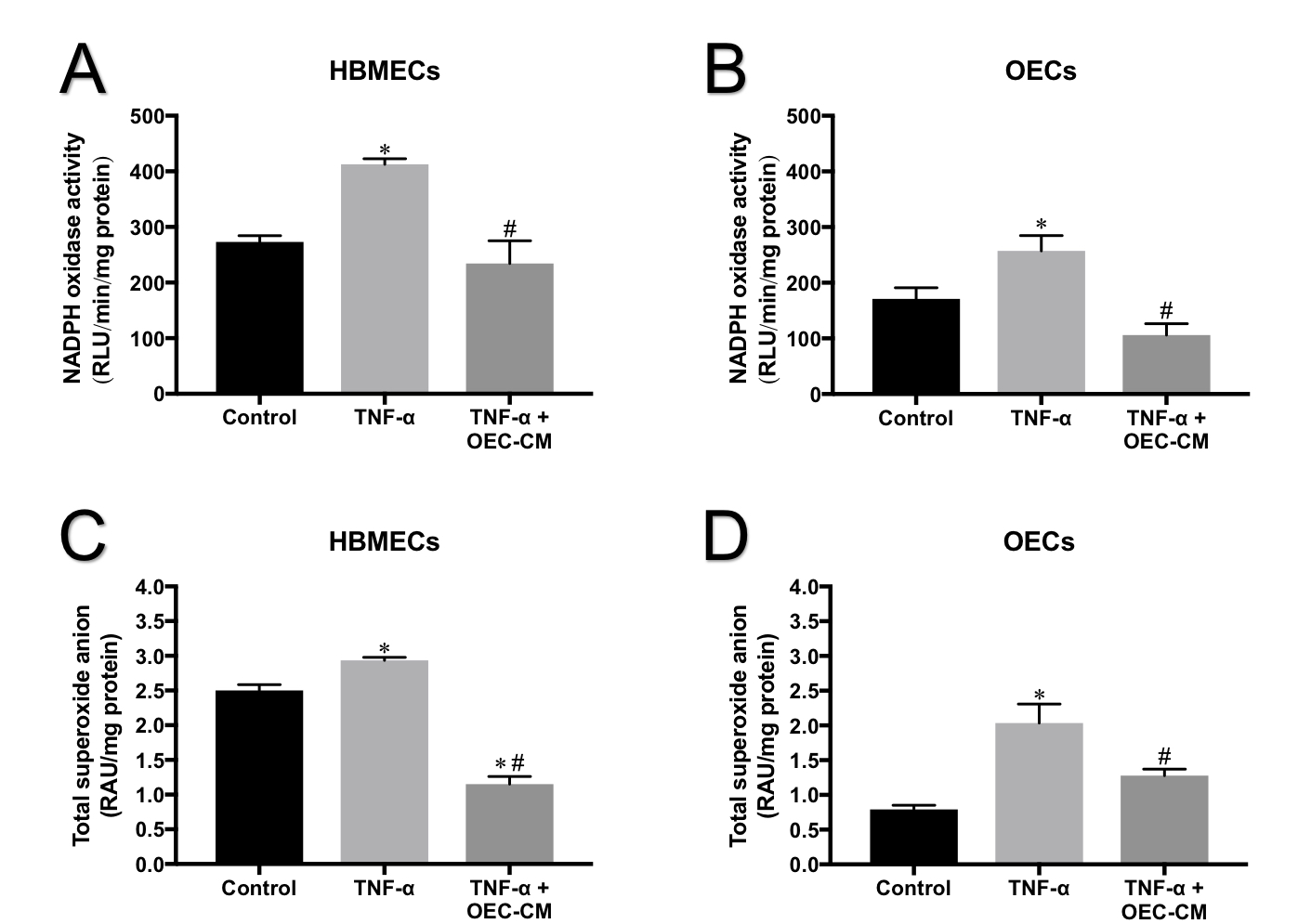

Supplement: Supplementary file 6 — Supplementary Fig. 5 (JPG 230 KB) [file 12015_2022_10439_MOESM6_ESM.jpg]

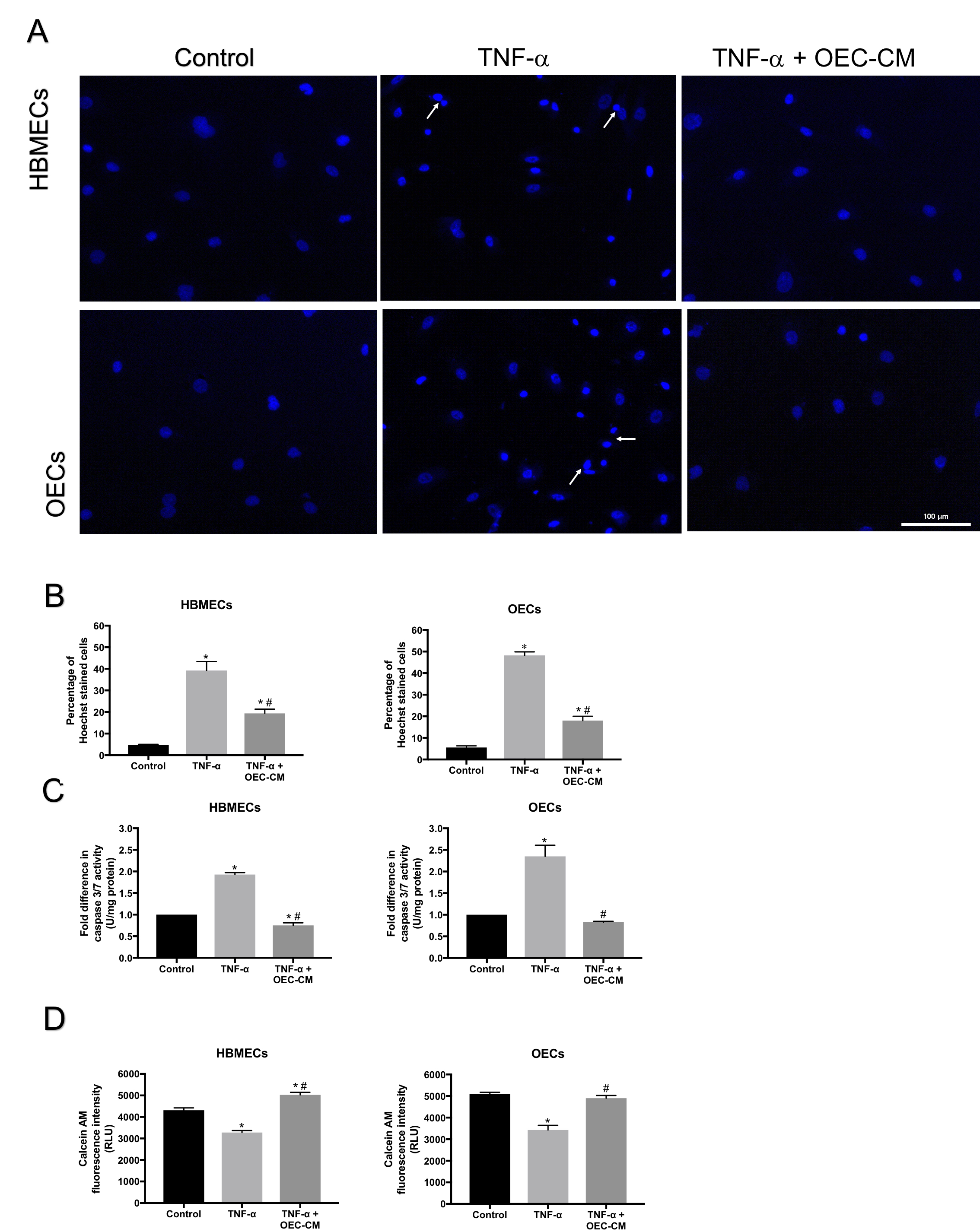

Supplement: Supplementary file 7 — Supplementary Fig. 6 (JPG 3687 KB) [file 12015_2022_10439_MOESM7_ESM.jpg]

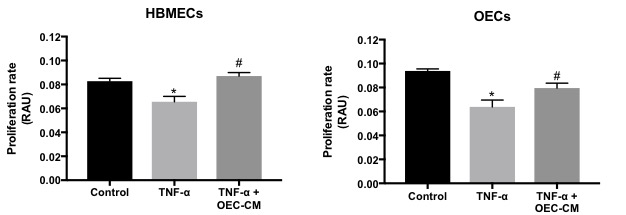

Supplement: Supplementary file 8 — Supplementary Fig. 7 (JPG 23 KB) [file 12015_2022_10439_MOESM8_ESM.jpg]

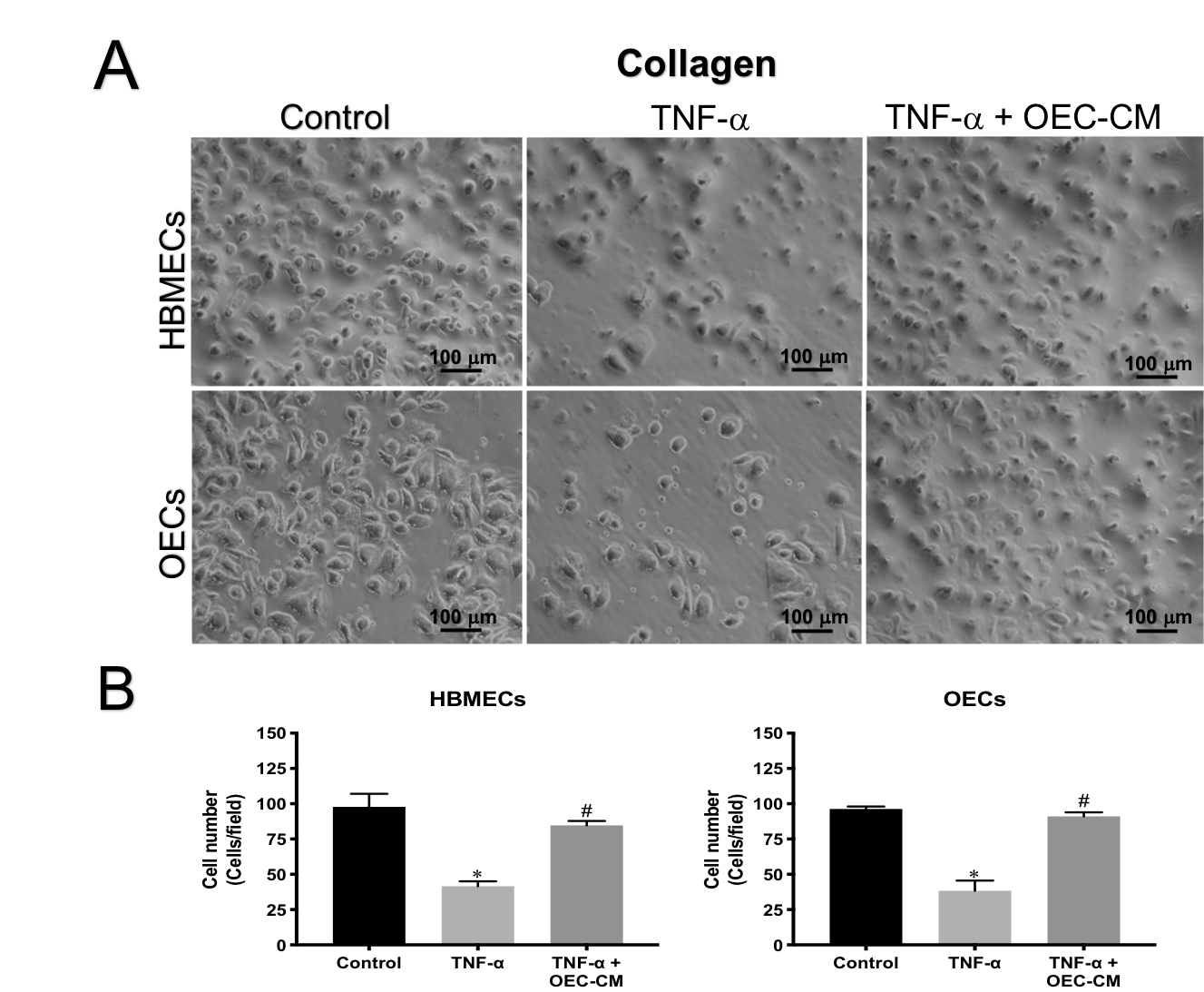

Supplement: Supplementary file 9 — Supplementary Fig. 8 (JPG 502 KB) [file 12015_2022_10439_MOESM9_ESM.jpg]

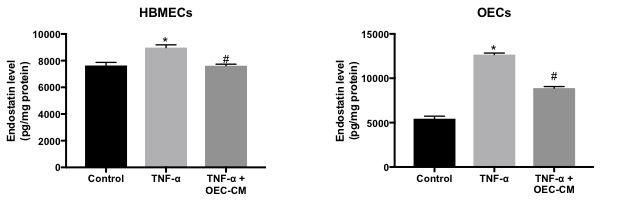

Supplement: Supplementary file 10 — Supplementary Fig. 9 (JPG 21 KB) [file 12015_2022_10439_MOESM10_ESM.jpg]
